# Potential Biomarkers for Feed Efficiency-Related Traits in Nelore Cattle Identified by Co-expression Network and Integrative Genomics Analyses

**DOI:** 10.3389/fgene.2020.00189

**Published:** 2020-03-04

**Authors:** Andressa O. de Lima, James E. Koltes, Wellison J. S. Diniz, Priscila S. N. de Oliveira, Aline S. M. Cesar, Polyana C. Tizioto, Juliana Afonso, Marcela M. de Souza, Juliana Petrini, Marina I. P. Rocha, Tainã F. Cardoso, Adhemar Zerlotini Neto, Luiz L. Coutinho, Gerson B. Mourão, Luciana C. A. Regitano

**Affiliations:** ^1^Center for Biological and Health Sciences, Federal University of São Carlos, São Carlos, Brazil; ^2^Department of Animal Science, Iowa State University, Ames, IA, United States; ^3^Embrapa Pecuária Sudeste, Empresa Brazileira de Pesquisa Agropecuária, São Carlos, Brazil; ^4^Department of Agroindustry, Food and Nutrition, Luiz de Queiroz College of Agriculture, University of São Paulo, Piracicaba, Brazil; ^5^NGS Genomics Solutions, Piracicaba, Brazil; ^6^Exact Sciences Institute, Federal University of Alfenas, Alfenas, Brazil; ^7^Embrapa Informática Agropecuária, Empresa Brazileira de Pesquisa Agropecuária, Campinas, Brazil; ^8^Department of Animal Science, Luiz de Queiroz College of Agriculture, University of São Paulo, Piracicaba, Brazil

**Keywords:** *Bos indicus*, feed efficiency, hub genes, systems biology, WGCNA

## Abstract

Feed efficiency helps to reduce environmental impacts from livestock production, improving beef cattle profitability. We identified potential biomarkers (hub genes) for feed efficiency, by applying co-expression analysis in *Longissimus thoracis* RNA-Seq data from 180 Nelore steers. Six co-expression modules were associated with six feed efficiency-related traits (*p*-value ≤ 0.05). Within these modules, 391 hub genes were enriched for pathways as protein synthesis, muscle growth, and immune response. Trait-associated transcription factors (TFs) *ELF1*, *ELK3*, *ETS1*, *FLI1*, and *TCF4*, were identified with binding sites in at least one hub gene. Gene expression of *CCDC80*, *FBLN5*, *SERPINF1*, and *OGN* was associated with multiple feed efficiency-related traits (FDR ≤ 0.05) and were previously related to glucose homeostasis, oxidative stress, fat mass, and osteoblastogenesis, respectively. Potential regulatory elements were identified, integrating the hub genes with previous studies from our research group, such as the putative cis-regulatory elements (eQTLs) inferred as affecting the *PCDH18* and *SPARCL1* hub genes related to immune system and adipogenesis, respectively. Therefore, our analyses contribute to a better understanding of the biological mechanisms underlying feed efficiency in bovine and the hub genes disclosed can be used as biomarkers for feed efficiency-related traits in Nelore cattle.

## Introduction

Due to the intensified demand for livestock products, there is a growing need for strategies to improve the efficiency of use of natural resources (Rojas-Downing et al., 2017). Among the strategies, it has been proposed to identify and select more feed-efficient animals, which can maintain the same production levels with less feed (Moore et al., 2009). This approach can minimize the environmental impact of livestock production through a reduction in greenhouse gas emissions and natural resources required for meat production (Basarab et al., 2003; Velazco et al., 2017) ensuring the beef production profitability (Basarab et al., 2003). Unfortunately, feed efficiency is impacted by a myriad of factors (Moore et al., 2009), it is measured late in life, and is an expensive phenotype to obtain.

Phenotypic and genomic variations have been reported for feed efficiency indexes (Basarab et al., 2003; Nkrumah et al., 2006; de Oliveira et al., 2014). Estimates of genomic heritability for feed efficiency measures ranged from 0.18 to 0.57 (de Oliveira et al., 2014) in Nelore cattle. Residual feed intake (RFI) has been proposed as the best choice for genetic selection because it has moderate heritability (de Oliveira et al., 2014) and independent of growth and body weight (BW) traits (Moore et al., 2009). Although it is possible to select efficient animals based on the RFI, it is still a challenging trait as multiple biological and physiological mechanisms impact the final phenotype (Nkrumah et al., 2006; Moore et al., 2009).

The genetic basis of feed efficiency has been explored in several studies revealing candidate genes, quantitative trait loci (QTLs) (de Oliveira et al., 2014; Olivieri et al., 2016) and enriched biological processes (Alexandre et al., 2015; Tizioto et al., 2015, 2016; Fonseca et al., 2019) in Nelore cattle. Tizioto et al. (2016) highlighted the enrichment of oxidative stress biological processes from 73 differentially expressed (DE) genes in muscle, comparing efficient and inefficient groups of Nelore steers. Alexandre et al. (2015) identified inflammatory response or inflammation-related ontology terms as over-represented from liver transcriptomic analysis in Nelore. Additionally, biological processes related to energy metabolism, protein turnover, and immune system have been highlighted as pivotal for feed efficiency (Alexandre et al., 2015; Tizioto et al., 2015, 2016; Mukiibi et al., 2018).

Previous studies investigating the genomic control of feed efficiency have not attempted to estimate the association of gene expression levels, or their effect, on feed efficiency phenotypes. Further, these studies have not evaluated the impact of gene interactions. Integrative approaches, such as systems biology methods, have been proposed to dissect complex phenotypes and overcome the limitations of single-data-type analysis (Alexandre et al., 2015; Weber et al., 2016; de Oliveira et al., 2018; Diniz et al., 2019; Fonseca et al., 2019). Among the integration approaches, grouping thousands of genes through co-expression networks analysis reduces the high-dimensionality of data and focuses on the relationship between a few modules (e.g., gene/transcript clusters) and the traits of interest (Langfelder and Horvath, 2008). Networks-based analyses identify the highly connected genes (i.e., hub genes), which may act as the main regulators of biologically meaningful pathways (Langfelder et al., 2013; van Dam et al., 2018).

Considering that there is more investigation to understand the complex biological architecture and to identify regulators underpinning feed efficiency, we adopted a weighted gene co-expression network analysis (WGCNA) framework to identify potential biomarkers (hub genes) modulating feed efficiency-related traits in Nelore cattle. Additionally, we investigated potential regulatory elements by integrating the hub genes with previous studies by our research group.

## Materials and Methods

### Phenotypic Data

All experimental procedures were conducted in accordance with the Institutional Animal Care and Use Committee Guidelines of the Brazilian Agricultural Research Corporation—EMBRAPA (CEUA Protocol 01/2013).

Phenotypes from 192 Nelore steers were previously described elsewhere (Cesar et al., 2018; de Lima, 2019). These steers were a subsample from a broader experimental population of 593 steers (de Oliveira et al., 2014) for which muscle RNAseq data were available. Briefly, the animals were offspring from 34 unrelated sires selected to represent the main Brazilian genealogies, according to the pedigree information obtained from the Brazilian Zebu Breeders Association. The phenotypic data were collected from cattle born in 2007, 2008, and 2009, finished at two different feedlots, Embrapa Pecuária Sudeste (São Carlos, SP, Brazil—feedlot 1) and Embrapa Gado de Corte (Campo Grande, MS, Brazil—feedlot 2). The animals were raised in a grazing system before entering the feedlots at an average age of 25 months. Upon entering the feedlot, steers underwent 28 days of acclimation, followed by at least 70 days of data collection on the same diet formulation (40% silage and 60% concentrate) fed twice daily.

The animals were evaluated for feed efficiency-related traits as described by de Oliveira et al. (2014), including average daily gain (ADG, kg/d), BW (kg), dry matter intake (DMI, kg/d), feed conversion ratio (FCR, feed intake/gain; kg/kg), feed efficiency ratio (FE, gain/feed intake; kg/kg), Kleiber index (KI, ADG/MBW; kg/kg), metabolic BW (MBW, kg), RFI (kg/d), and relative growth rate (RGR,%/d).

### RNA Sequencing and Data Processing

A total of 192 samples from the *Longissimus thoracis* muscle of Nelore steers were used in this study. The RNA sequencing from these samples was performed at the Genomics Core Facility at ESALQ (Piracicaba, SP, Brazil), as previously described elsewhere (Cesar et al., 2018) and archived on the European Nucleotide Archive (ENA) under accessions: PRJEB13188, PRJEB10898, and PRJEB19421.

The data quality control (QC), alignment, and quantification were previously performed and described elsewhere (Cesar et al., 2018). In summary, the data QC of raw reads was performed by FastQC v.0.11.2 (Andrews, 2010) and MultiQc v.1.4 (Ewels et al., 2018). The paired-end reads were filtered using the Seqyclean v1.4.13 package^1^, which removed all the reads with a mean Phred quality score lower than 24. Reads shorter than 65 base pairs (bp), as well as primers and vector contaminants, were removed using information from the UniVec database^2^.

Sequencing reads were aligned to *Bos taurus* genome assembly (UMD3.1 assembly available in the Ensembl database) to generate expression values using the RSEM (RNA-Seq by expectation maximization) (Li and Dewey, 2011) software. The same software also performed the quantification and normalization of transcripts mapped per million bases of the genome [transcripts per million (TPM)]. An average of 16 million reads was mapped with a rate of 86% unique mapping per sample.

### Co-expression Network and Module Trait Association (MTA) Analyses

For co-expression network analysis, we used the R package WGCNA as described by Langfelder and Horvath (2008). Samples were filtered out from the analysis if they were missing phenotypes for all traits. Additionally, genes with less than two counts in 90% of the samples were excluded (6051 genes out of 14,997 expressed genes in our muscle samples). The TPM values were logarithmically transformed [*log*_*2*_ (TPM + 1)] and a linear model was fitted to adjust the gene expression data (TPM values) for the batch effect of sequencer lane by using the R package Limma v.3.36.2 (Ritchie et al., 2015).

From the adjusted expression data, we constructed a signed co-expression network based on Spearman correlation. Considering the scale-free topology criteria, we chose a soft thresholding power β = 16, that satisfied the scale-free topology assumptions (linear regression model fitting index *R*^2^ = 0.81). Genes were clustered into modules based on the Topological Overlap Measure (TOM) by applying the dynamic tree cut v.1.63.1 package. The module eigengene (ME), i.e., the value of the first principal component of each module, was estimated and used to associate the modules to each trait. To this end, we fitted a linear model that included the contemporary group (CG) as a fixed effect and animal’s age at slaughter as a covariate, using the lm function available in R. The CG was described previously elsewhere (de Oliveira et al., 2014) and included the significant environmental effects as feedlot location, year of the experiment, animal origin, and pen type. The environmental effects were previously tested (de Oliveira et al., 2014) using the MIXED procedure (SAS Institute Inc, 2010), and the significant fixed effects for at least one trait were included in a common CG for all traits. The mixed linear model included the MEs (*n* = 25), taken as the dependent variable, and the phenotypes: ADG, BW, DMI, FCR, FE, MBW, KI, RFI, RGR represented as follows:

$$y_{i\,j}=\mu +C\,G_{i}+b_{1}\,\left(A_{i\,j}-\bar{A}\right)+b_{2}\,\left(T_{i\,j}-\bar{T}\right)+\varepsilon_{i\,j},$$

where:

*y*_*ij*_ are the ME values for the *j*th animal for the *i*th CG;

μ is the mean;

*b*_1_,*b*_2_ are the regression coefficients associated with the animal’s age at slaughter and the observation of phenotype, respectively;

*CG*_*i*_ is the fixed effect of the CG;

*A*_*jj*_ is the animal’s age at slaughter for the *j*th animal for the *i*th CG; $\bar{A}$ is the mean age at slaughter;

*T*_*ij*_ is the observation of the phenotype for the *j*th animal for the *i*th CG; $\bar{T}$ is the mean phenotype;

ε_*ij*_ is the random residual effect [∼ *N* (0, σ^2^_e_)].

Modules significantly associated with feed efficiency-related traits (*p*-value ≤ 0.05) were selected for further analyses.

### Hub Gene Identification and Pathway Over-Representation Analysis

We used two approaches to identify putative regulatory genes and their effect on feed efficiency-related traits. First, we selected the highly interconnected genes (hub genes) within the associated modules based on module membership (MM) ≥ 0.8 (Langfelder and Horvath, 2008). Second, we used the previously mentioned linear model to associate adjusted expression from the selected hub genes with the phenotypic traits. However, in these analyses, the phenotypes, instead of ME, were taken as the dependent variable. Further, the hub genes’ normalized expression (TPM) was adjusted for the CG, animal’s age at slaughter, and lane effect. The hub genes with FDR ≤ 0.05 (Benjamini and Hochberg, 1995) were considered significant.

To gain biological insights into the genetic factors impacting feed efficiency, we performed an over-representation pathway analysis for the identified hub genes using ClueGO v.2.5.1 (Bindea et al., 2009) based on the *Bos taurus* database. In the enrichment analysis, the redundant terms were clustered, considering the kappa score = 0.4 and the *p*-values corrected with the Benjamin–Hochberg test. Only the pathways with FDR ≤ 0.05 were considered. The Cytoscape software (Shannon et al., 2003) was used for network construction and data visualization.

### Transcription Factor Binding Site (TFBS) Enrichment Analysis of Hub Genes

To identify potential transcription factors (TFs) acting as regulators among the hub genes, we carried out a TF binding site (TFBS) enrichment analysis using the Rcis Target software (Aibar et al., 2017). This software identified TFBS motifs over-represented in our list of hub genes. Additionally, we used a human database, scoring 500 bp upstream of transcription start site (TSS), and TFBS motifs with normalized enrichment score (NES) > 3.0 were considered as significantly associated to TFs. Only the TFs included in the bovine manually curated database (de Souza et al., 2018) and expressed in our muscle samples were selected. TFs predicted as regulators of hub genes belonging to at least two modules associated with feed efficiency-related traits were categorized according to biological processes by using the PANTHER database^3^. Data visualization was carried out on Cytoscape software (Shannon et al., 2003).

### Data Integration Analysis

To further identify putative regulatory elements linked to the hub genes, we built a gene list containing the genes reported in previous studies using the same population, with genes identified as DE in muscle between efficient and inefficient groups (*n* = 73) (Tizioto et al., 2016), QTLs associated with feed efficiency-related traits (*n* = 36) (de Oliveira et al., 2014), genes reported in eQTL analysis (*n* = 1643 genes, 1268 cis and 10,334 trans-eQTLs) (Cesar et al., 2018), and TFs described for bovine (*n* = 865) (de Souza et al., 2018). Genes identified in the 1Mb QTL windows reported by de Oliveira et al. (2014) were retrieved using the R package Biomart version 3.5 (Durinck et al., 2009). For eQTLs, we considered the list of genes that were regulated by each eQTL. This gene list was used to retrieve additional features for the hub genes found in this study. Cytoscape software (Shannon et al., 2003) was used for data visualization.

## Results

**FIGURE 1:**
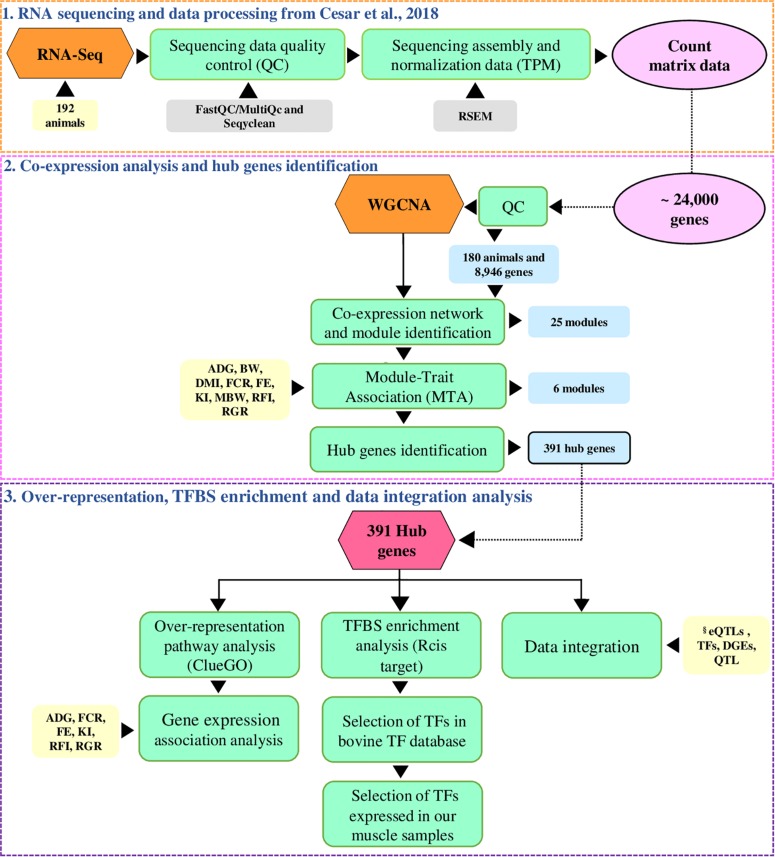
Overview of the analysis. The analysis workflow is separated into three parts. (1) The orange dashed box corresponds to the RNA sequencing and data processing performed by Cesar et al. (2018). (2) The pink dashed box corresponds to the co-expression network construction and the hub genes identification. (3) The purple dashed box corresponds to the over-representation, transcription factor binding site (TFBS) enrichment analysis, and data integration analysis. The steps taken are described in the green boxes, the count matrix represented by the pink circle, the yellow boxes are the input, the light blue boxes are the output in each step, and the gray boxes are the tools used in each step. ADG = average daily gain; BW = body weight; DMI = dry matter intake; FCR = feed conversion ratio, FE = feed efficiency ratio; KI = Kleiber index, MBW = metabolic body weight, RFI = residual feed intake, RGR = relative growth ratio. §Database used in our study derived from eQTL analysis (Cesar et al., 2018); manually curated transcription factors (TFs) bovine database (de Souza et al., 2018); differentially expressed genes (DEGs) in muscle for RFI (Tizioto et al., 2016); feed efficiency QTL (de Oliveira et al., 2014).

As illustrated in Figure 1, we applied the co-expression network (WGCNA) approach to identify the most relevant genes (hub genes) in co-expression clustered genes (modules) associated with feed efficiency-related traits. We also integrated the hub genes with previously published results obtained by our research group to point out regulatory elements underlying the hub genes.

### Gene Expression and Phenotypic Data

After QC, we kept 8946 genes expressed in the *Longissimus thoracis* muscle from 180 Nelore steers (Supplementary Table S0). The descriptive statistics for the nine feed efficiency-related traits used in this study are presented in Table 1.

**TABLE 1:** Mean, standard deviation (SD), minimum (Min), and maximum (Max) observed values for feed efficiency-related traits in Nelore cattle.

**Traits^a^**	**Mean ± SD**	**Min**	**Max**
ADG (kg/d)	1.42 ± 0.29	0.80	2.33
BW (kg)	382.98 ± 51.02	280.5	515.5
DMI (kg/d)	8.56 ± 1.23	5.40	12.18
FCR (feed intake/gain; kg/kg)	6.24 ± 1.40	3.74	11.50
FE (gain/feed intake; kg/kg)	0.17 ± 0.04	0.086	0.27
KI (ADG/MBW; kg/kg)	0.016 ± 0.004	0.008	0.027
MBW (kg)	86.43 ± 8.6	68.54	108.19
RFI (kg/d)	−0.028 ± 0.67	−1.71	1.81
RGR (%/d)	0.17 ± 0.04	0.09	0.30

*^*a*^The sample size for all feed efficiency-related traits described in this table was 180 steers. ADG: average daily gain; BW: body weight; DMI: dry matter intake; FCR: feed conversion ratio; FE: feed efficiency ratio; KI: Kleiber index; MBW: metabolic body weight; RFI: residual feed intake; RGR: relative growth ratio.*

### Co-expression Network and MTA Analyses

Based on the WGCNA framework, we identified 25 modules (Supplementary Figure S1). The MEs were able to explain from 34% (MEblue) to 67% (MEgray60) of the gene expression variation (Supplementary Table S1).

Six co-expressed modules significantly associated with feed efficiency-related traits were selected for further analysis (MTA, *p*-value ≤ 0.05) (Supplementary Figure S2). The MEbrown and MEyellow modules exhibited the highest number of associated traits, including ADG, FCR, FE, KI, and RGR. The traits FCR and RGR had a greater number of associated modules, with four and five, respectively (Table 2). Further, The ME purple was exclusively associated with RFI.

**TABLE 2:** Description of significantly associated gene co-expression modules with feed efficiency-related traits in Nelore cattle.

**Module**	**Traits (coefficient)^a^**	**^b^Gene**	**^c^ME (%)**
MEblack	RGR (0.5)	309	52.9
MEbrown	FCR (−0.01), FE (0.5), ADG (0.05), KI (5), RGR (0.6)	1125	34.8
MElightcyan	FCR (−0.01), KI (4), RGR (0.5)	507	40.4
MEpurple	RFI (−0.02)	224	35.1
MEtan	FCR (−0.01), FE (0.4), RGR (0.5)	92	62.8
MEyellow	FCR (−0.02), FE (0.6), ADG (0.06), KI (6), RGR (0.6)	732	39.6

*^*a*^The coefficient from linear model associated with the traits, presented in parentheses (*p*-value ≤ 0.05); ^*b*^Number of genes clustered into the module; ^*c*^ME variation = expression variation explained by the module eigengene; ADG: average daily gain; BW: body weight; DMI: dry matter intake; FCR: feed conversion ratio; FE: feed efficiency ratio; KI: Kleiber index; MBW: metabolic body weight; RFI: residual feed intake; RGR: relative growth ratio.*

### Hub Gene Association and Pathway Over-Representation Analyzes

A total of 391 hub genes were identified based on MM ≥ 0.8 within the six trait-associated modules (Table 2). We also found association (FDR ≤ 0.05) among hub genes and the traits ADG (*n* = 5), FCR (*n* = 186), FE (*n* = 147), KI (*n* = 137), and RGR (*n* = 278) (Supplementary Tables S2, S3).

**FIGURE 2:**
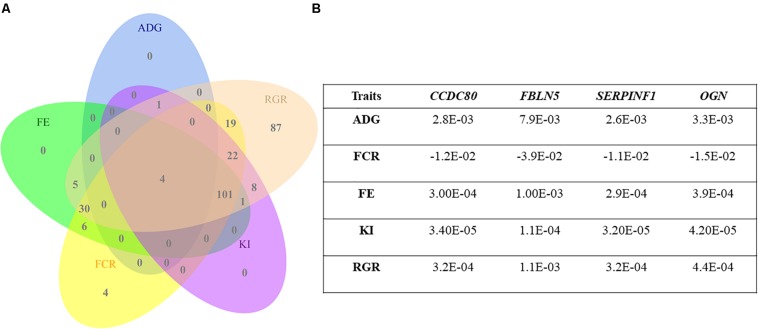
**(A)** Venn diagram showing the number of hub genes whose expression levels were associated with feed efficiency-related traits (*CCDC80*, *FBLN5*, *SERPINF1*, and *OGN*). **(B)** The table to the right describes the coefficients obtained from a linear model for the hub genes’ expression overlapping simultaneously average daily gain (ADG), feed conversion ratio (FCR), feed efficiency ratio (FE), Kleiber index (KI), and relative growth ratio (RGR).

The expression levels of *CCDC80*, *FBLN5*, *SERPINF1*, and *OGN* genes were identified as simultaneously impacting the five traits described in Figure 2. Although the MEpurple module was associated with RFI, the hub genes within this module were not individually associated with this trait.

**FIGURE 3:**
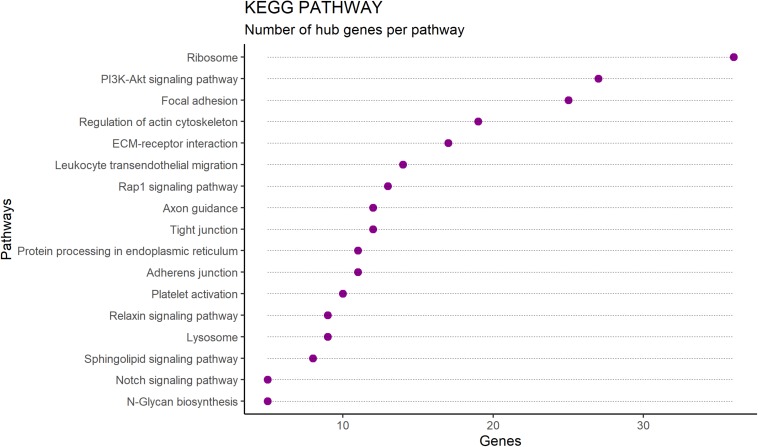
Number of hub genes per pathway identified by enrichment analysis performed by ClueGO software on RNAseq data from Nelore *Longissimus thoracis*. Enriched pathways are shown on the *Y*-axis and the number of hub genes per pathway is shown on the *X*-axis.

**FIGURE 4:**
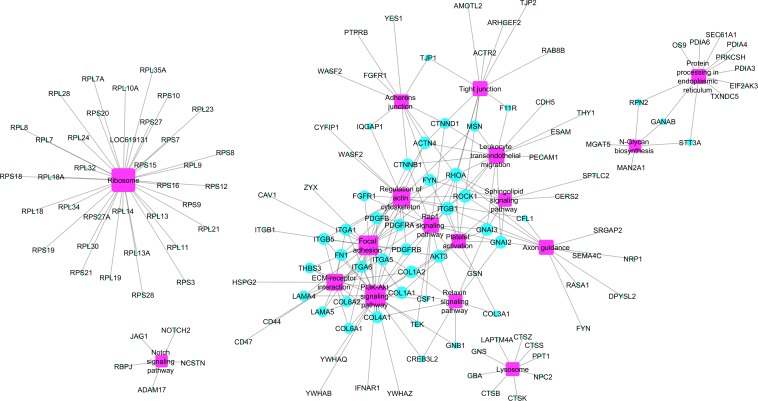
Network hub genes and enriched pathways identified by ClueGO software. The size of the purple squares (pathways) represents the connectivity degree of the nodes (turquoise circles representing hub genes) based on the number of connections to the pathways.

Enriched pathways identified for the 391 hub genes (Supplementary Table S4) are shown in Figure 3 and included the ribosome pathway (highest number of genes) and the notch signaling pathway and junction-glycan biosynthesis (tied for the smallest number of genes) (adj *p* < 0.05). Many of the hub genes identified encode proteins involved in multiple pathways, as demonstrated in Figure 4.

### TFBS in Hub Genes

A total of 299 TFs described for bovine and expressed in our muscle samples was identified as having TFBS within the hub genes (Supplementary Tables S5, S6).

**FIGURE 5:**
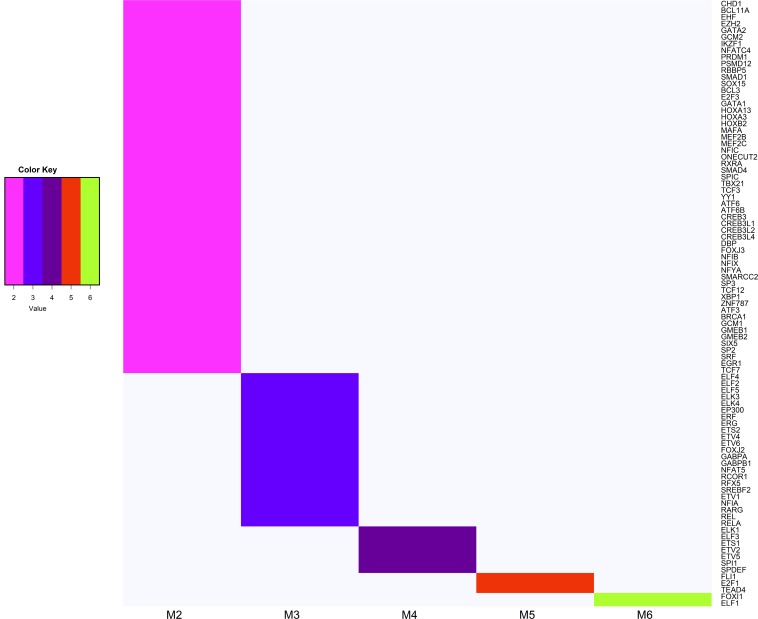
Heatmap of transcription factors (TFs) that are potential regulators of hub genes belonging to at least two modules associated with feed efficiency-related traits. The TF gene names shown on the *Y*-axis, and the number of modules that the TFs interact is shown on the *X*-axis (M2 shown on pink = TFs interacting with two modules; M3 shown in blue = TFs interacting with three modules; M4 shown in purple = TFs interacting with four modules; M5 shown in red = TFs interacting with five modules; M6 shown in green = TFs interacting with six modules).

Within this subset, 91 TFs were expected to interact with hub genes belonging to at least two modules associated with feed efficiency-related traits (Figure 5). Among these TFs, we highlight *E2F1*, *FLI1*, *TEAD4* which interact with hub genes within five modules, and *ELF1*, *FOXI1* TFs interacting with hub genes within six modules. According to PANTHER, 40% of ninety-one TFs were categorized into biological processes related to metabolism (Supplementary Figure S3).

**FIGURE 6:**
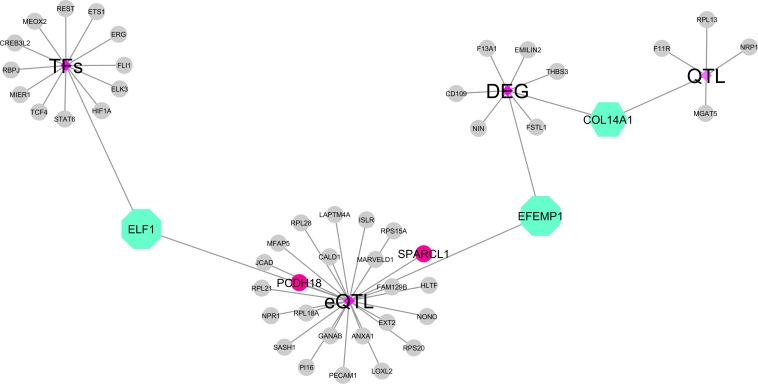
The integrative data network of the hub genes identified in the present study with data from previous functional studies. Shown in the figure are transcription factors, annotation as transcription factors, genes affected by expression quantitative trait locus (eQTLs), and genes previously associated with feed efficiency (differentially expressed genes and QTLs). The purple diamonds represent the information from previous studies, the green octagons are the hub genes from the present study that were also identified in more than one study, the gray circles novel are hub genes from the present study, and the pink circles are the hub genes affected by cis-eQTLs.

**FIGURE 7:**
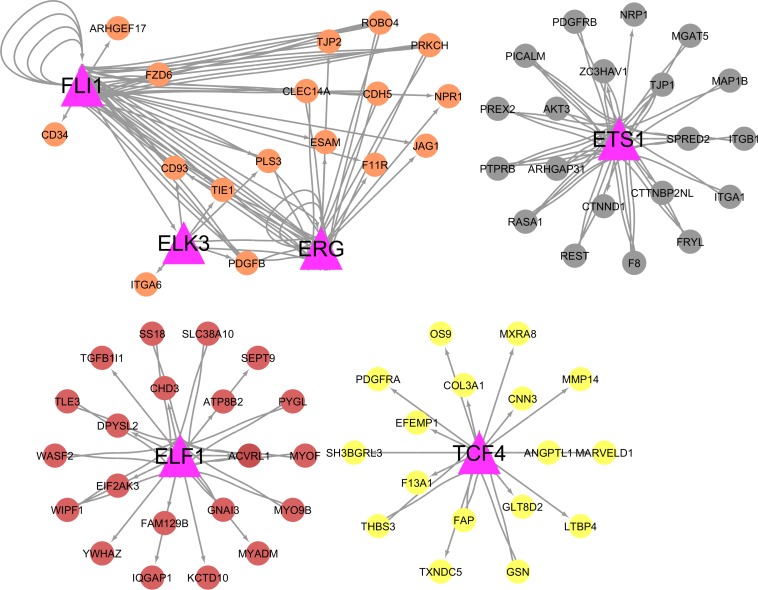
Integration of the transcription factors (hub-TFs) with their targets. Pink triangles represent hub-TF genes. The colors of the nodes are based on the module for which the hub-TF belongs.

Thirteen out of 391 hub genes identified in this study were themselves TFs (hub-TFs) (Figure 6). Based on TFBS enrichment analysis, among these hub-TFs, six were potential regulators for multiple hub genes identified within this study (Figure 7). All but one (*ERG*) of these hub-TFs expression levels were significantly associated with at least one of the feed efficiency-related traits (FDR ≤ 0.05). These hub-TFs included: *ELF1*, associated with RGR, *ELK3*, associated with FCR, FE, KI, and RGR, *ERG* (no association), *ETS1*, associated with FCR, FE, KI, and RGR, *FLI1*, associated with FCR and RGR, and *TCF4*, associated with FE, KI, and RGR (Supplementary Table S3).

### Data Integration Analysis

An integrative analysis using genes identified in published results from our research group was conducted to determine which hub genes reported here were previously associated with feed efficiency-related traits as QTLs (de Oliveira et al., 2014), as DE genes in contrasting RFI groups (Tizioto et al., 2016), as well as putative regulatory elements (Cesar et al., 2018) and TFs described for bovine (de Souza et al., 2018).

Among the hub genes, 26 were linked to eQTLs, like the TF *EFL1* and the gene *EFEMP1*, which were affected by putative trans-regulatory elements (trans-eQTLs). We also observed hub genes described as affected by cis-eQTL regions, including the *PCDH18* and *SPARCL1.* Interestingly, the hub gene *COL14A1* was identified as DE for RFI and located in a QTL associated with maintenance efficiency and partial efficiency of growth traits (Figure 6).

## Discussion

In this study, we used a co-expression network analysis using muscle transcriptome data to identify potential biomarkers (hub genes) associated with feed efficiency-related traits in Nelore steers. The role of hub genes was investigated based on biological functions underlying feed efficiency, as well as their connection to putative regulatory elements. Muscle hypertrophy was one such biological process of interest, which influenced by protein turnover activated through signaling pathways, depending on physiological and pathological conditions (Costamagna et al., 2015). Accordingly, some of the hub genes identified underlie pathways related to protein synthesis, muscle growth, and immune response. We also identified putative regulatory elements linked to the hub genes associated with feed efficiency-related traits. Within the regulatory elements are TFs described as involved in glucose homeostasis, fat mass, and energy balance, making them excellent candidate regulators of feed and growth efficiency traits. From our results, FCR and RGR seem to be controlled by genes involved in a greater variety of biological processes than other feed efficiency-related traits. This fact could be related to the complex biological architecture of FCR and RGR, which are known to be correlated to BW, compared for example, to RFI, which is a more specific trait, reflecting mainly the ideal energy necessary to for weight gain and in our study was associated only with one module (purple).

### Immune System

The pathway enrichment analysis of co-expressed transcripts revealed hub genes related to immune response, i.e., adherens junctions, leukocyte transendothelial migration (TEM), regulation of actin cytoskeleton, sphingolipid signaling pathway, and tight junctions (adj *p* ≤ 0.05).

Several proteins encoded by the hub genes were identified here as taking part simultaneously in many pathways, as *RHOA* and *ROCK*. RhoA is a small Rho-GTPases (Schimmel et al., 2017) associated with Rho-associated kinases (ROCKs), which are downstream targets of RhoA (Mokady and Meiri, 2015). The proteins encoded by these genes are pivotal in the regulation of the actin cytoskeleton, where RhoA activity was reported to be influenced by extracellular signals (Schimmel et al., 2017).

Regulation of the actin cytoskeleton is essential to endothelial cell junction stability, vascular permeability (Radeva and Waschke, 2018), and leukocyte migration (Marelli-Berg and Jangani, 2018). In our results, the *ROCK* and *RHOA* gene expression levels were associated with RGR, being the *RHOA* also associated with FCR, FE, and KI. These genes encode proteins that not only take part in the regulation of the actin cytoskeleton but also appear in the enriched pathways adherens junctions and tight junctions, indicating that these pathways are linked with each other. The adherens and tight junctions are endothelial cell junctions, which play a role in the endothelial cell barrier and are connected to the actin cytoskeleton through different molecules (Cerutti and Ridley, 2017). Additionally, endothelial cell barriers are essential to avoid organ dysfunction (Radeva and Waschke, 2018).

Hub genes related to immune response processes were also identified, such as *PECAM1*, *GNAI2*, and *GNAI3*. The PECAM1 protein, whose gene was identified in this study as associated with RGR, participates in the leukocyte TEM pathway. Further, this protein also is known to limit cellular activation responses in circulating platelets and leukocytes and is required for leukocyte transmigration (Privratsky and Newman, 2014). The *GNAI2* and *GNAI3* genes encode proteins assigned to the sphingolipid signaling pathway in our enrichment analysis, both with an association with a negative effect on FCR and an association with a positive effect on FE and RGR in our linear regression analysis. These genes are members of Guanine nucleotide-binding protein (G proteins) family and code for part of the subunit Gαi (Zhang et al., 2019), which functions through sphingosine-1-phosphate (S1P) receptors (S1P1-5) to increase the endothelial barrier permeability (Reinhard et al., 2017).

Based on the over-represented pathways and the hub genes associated with feed efficiency-related traits being involved in the immune system, we suggest that a fine regulation of endothelial permeability and leukocyte migration underlie feed efficiency variation. The role of the immune system-related biological processes in feed efficiency in Nelore cattle was previously reported by Alexandre et al. (2015), who found the immune response over-represented in inefficient Nelore as an outcome of increased liver injury. Accordingly, different immune response processes were associated with feed efficiency in steers from the same population studied here (de Oliveira et al., 2018). Additionally, a better adaptive immune response was considered important in more feed efficient animals since they require less feed to support their immune systems, which might free up energy for muscle growth as compared to inefficient animals (Horodyska et al., 2018b).

### Protein Synthesis and Muscle Growth

Animal feed efficiency is directly related to energy usage toward protein turnover and fat metabolism (Herd and Arthur, 2009). Among the biological mechanisms acting and regulating these processes, we identified ribosome and PI3K/AKT pathways as over-represented. Based on the assumption that inefficient animals require more maintenance energy (Moore et al., 2009; Khiaosa-ard and Zebeli, 2014), we suggest that increased expression of ribosomal genes may be favorable to promote improved feed efficiency. The efficiency of translation affects protein synthesis rate, which is directly impacted by the number of ribosomes (Bush et al., 2003). In pigs, protein synthesis efficiency was reported as impacting muscle growth (Horodyska et al., 2018a) and higher ribosomal gene expression levels were identified as beneficial to feed efficiency (Gondret et al., 2017). In a previous study with contrasting RFI groups from the same population analyzed here, DE genes related to translational regulation and ribosome biosynthesis were described (Tizioto et al., 2016).

The PI3K pathway, together with AKT and mTOR, modulate muscle hypertrophy through the regulation of protein translation, where ribosome activity is essential (Davis et al., 2012). In this pathway, the proteins encoded by *AKT3* and *FGFR1*, hub genes identified in this analysis, have an essential role in the regulation of cell proliferation, differentiation, and migration. The three pathways are insulin-mediated and AKT activation leads to an increased number of myofibrils (Davis et al., 2012). Accordingly, a positive relationship between gene expression and the traits FE and RGR was observed for both genes, being the *FGFR1* expression also associated with higher KI.

### Energy Balance

Glucose is a pivotal source of cellular energy from food digestion (Yang, 2014). In this study, we identified expression of the hub genes associated with multiple feed efficiency-related traits involved in glucose homeostasis, e.g., the *CCDC80* gene, a potential negative regulator of adipogenesis whose dysfunction can lead to excessive body fat in mice (Grill et al., 2017). The hub gene *OGN* encodes a putative humoral anabolic bone factor protein produced in muscle tissue (Kaji, 2014) and previously identified as DE in liver samples from pigs divergent for RFI (Vigors et al., 2019). *OGN* plays a role in whole-body glucose homeostasis and energy balance (Lee et al., 2018) and is important in bone formation, helping to regulate the balance between osteoblastogenesis and adipogenesis with a positive effect on bone mass (Chen et al., 2017). In cattle, bone weight has been positively correlated with feed efficiency and negatively correlated with subcutaneous and intramuscular fat (Mader et al., 2009). In this study, *OGN* showed the effects on five traits (ADG, FCR, FE, KI, RGR), all with favorable associations, i.e., increased expression related to greater efficiency.

### Oxidative Stress

Among hub genes associated with multiple feed efficiency-related traits, *FBLN5* and *SERPINF1* were previously reported to be involved in oxidative stress and reactive oxygen species (ROS) production (Spencer et al., 2005; Ma et al., 2018). In this study, the *FBLN5* gene showed an effect on feed efficiency-related traits. *FBLN5* encodes a protein that may regulate the production of ROS in the vascular wall and act to protect endothelial dysfunction (Spencer et al., 2005). *SERPINF1* was identified as associated with the same five traits feed efficiency-related traits. *SERPINF1* encodes the pigment epithelium-derived factor (PEDF) protein (Li et al., 2016). This gene was previously reported as DE in divergent groups for RFI in livestock animals, chicken liver (Zhang et al., 2016), and pig blood (Jégou et al., 2016). The PEDF inhibits endothelial cells and can suppress NADPH oxidase-mediated ROS production in the cells, having potent anti-inflammatory, anti-oxidant, anti-angiogenic, and anti-thrombotic effects (Li et al., 2018). The PEDF is also described as a negative regulator of fat mass (Ma et al., 2018). The relationship between oxidative stress and fat mass was reported in a study with mice, where the increment in oxidative stress was related to obesity induced by diet (Marín-Royo et al., 2019).

In Nelore cattle, it was recently described that efficient animals seem to have a greater adaptation capacity to oxidative stress, demanding less energy expenditure than inefficient animals (Fonseca et al., 2019). Oxidative stress was previously associated with feed efficiency in our experimental population (Tizioto et al., 2015, 2016; de Oliveira et al., 2018), with genes related to oxidative stress over-represented in inefficient animals (Tizioto et al., 2016).

### Regulatory Elements

From the motif enrichment and integrative analysis results, potential cis- and trans-regulatory elements linked to feed efficiency were identified. Based on TFBS motifs analysis, 91 TFs were predicted to interact with hub genes belonging to at least two modules associated with feed efficiency-related traits. Among these TFs, 40% were categorized into metabolic processes by PANTHER software. Better metabolic processes efficiency was reported in animals with high feed efficiency (Mukiibi et al., 2018; Fonseca et al., 2019).

Thirteen hub genes were annotated as TFs described in cattle (hub-TFs). Among them, six hub-TFs (*ETS1*, *ELF1*, *ELK3*, *ERG*, *FLI1*, and *TCF4*) were predicted as putative regulators of many hub genes identified in this study and all except for *ERG*, had their increased expression levels associated with higher efficiency for at least one trait. The *ETS1*, *ELF1*, *ELK3*, *ERG*, and *FLI1* hub-TFs are members of the ETS family that encode TFs that play a role in vascular inflammation (Oettgen, 2006). The *ETS1* gene was previously pointed out as a potential regulator for feed efficiency in Angus cattle (Weber et al., 2016). The function of ETS1 impacts cancer cell viability resulting from reduced levels of glucose uptake and ATP production based on studies in *ETS1* knockouts mice (Zhang et al., 2017). These data indicate multiple potential roles for *ETS1* in biological processes impacting feed efficiency.

The *TCF4* (*aka TCF7L2*) was another important hub-TF also previously associated with feed efficiency in pigs (Fan et al., 2010). This TF was described with an important role in the Wnt signaling pathway (Feng et al., 2017), myogenic development (Anakwe, 2003), vascular smooth muscle cell proliferation (Zhuang et al., 2016), and the TGF-β signaling pathway (Contreras et al., 2016). In this study, the TCF4 was predicted to regulate the hub genes *F13A1*, *EFEMP1*, and *THSB3*, previously identified as DE for RFI in muscle samples from the same population (Tizioto et al., 2016).

The hub-TFs identified here, *ETS1* and *TCF4*, are connected to the FOXO1 pathway as regulators of *FOXO1* signaling in the liver (Oh et al., 2013; Li et al., 2019). The *FOXO1* pathway regulates whole-body energy balance and plays a role in oxidative stress, protein turnover, immunity modulation, glucose homeostasis, mitochondrial function, AKT signaling, mTOR signaling, WNT signaling, and insulin signaling (Cheng et al., 2010; Guo, 2014; Sanchez et al., 2014; Xing et al., 2018).

The ETS1 TF is as an important co-regulator of *FOXO1* during the fasting to fed state transition (Li et al., 2019). Based on this, we suggest that ETS1 plays a role in energy homeostasis in muscle and can be a major regulator of feed intake and growth efficiency traits in Nelore cattle. Unfortunately, we were not able to address this effect in our experiment, as the expression of *FOXO1* did not overcome data QC. Given these previous findings, it is plausible that *ETS1* and *TCF4* may be central regulators of energy metabolism in Nelore cattle, impacting variability in feed efficiency.

To search for SNPs affecting the expression of putative regulatory genes, we integrated the information on potential cis-regulatory and trans-regulatory elements previously described by our research group (Cesar et al., 2018). Among the 26 hub genes likely affected by eQTLs, *PCDH18* and *SPARCL1* were the only ones under control of cis-regulatory elements (cis-eQTL). The *PCDH18* gene is a member of the cadherin family (Aamar and Dawid, 2008) and encodes a protein involved in cell adhesion and the immune system (Vazquez-Cintron et al., 2012). The SPARCL1 protein controls adipogenesis in humans (Meissburger et al., 2016).

Some hub genes were identified as possible affected by trans-eQTL. *EFEMP1* was predicted as affected by one trans-eQTL located on chromosome 24 (BTA24) and was previously identified as a DE gene in muscle for RFI (Tizioto et al., 2016). Additionally, the hub-TF *ELF1* was predicted to be affected by five trans-eQTLs located on chromosome five (BTA5). Additionally, *ELF1* was predicted to interact with hub genes belonging to all modules (six modules) associated with feed efficiency-related traits. Finally, this hub-TF was previously described to have a potential regulatory effect on growth in pigs (Puig-Oliveras et al., 2014).

## Conclusion

Our co-expression approach revealed several hub genes and potential regulators expressed in skeletal muscle of Nelore steers modulating feed efficiency-related traits. By adopting a linear regression approach, we were able to estimate the impact of these hub genes expression levels on each trait. Some of these hub genes seem essential in muscle protein synthesis, energy balance, and immune response. By integrating eQTL data from the same population, we also revealed genomic regions with potential to regulate these hub genes’ expression, thus providing insights for the search of causative mutations. Future studies in other populations are necessary to confirm these potentials biomarkers for feed efficiency in *Bos indicus*.

## Data Availability Statement

The datasets generated for this study can be found in the European Nucleotide Archive (ENA)/PRJEB13188, PRJEB10898, and PRJEB19421.

## Ethics Statement

The animal study was reviewed and approved by the Institutional Animal Care and Use Committee Guidelines of the Brazilian Agricultural Research Corporation—EMBRAPA (CEUA Protocol 01/2013).

## Author Contributions

AL, JK, WD, PT, LC, and LR conceived the idea of this study. AL, JK, WD, AC, MS, JP, GM, and AN performed the bioinformatics and data analysis. AL, JK, WD, LR, PT, PO, AC, TC, LC, MR, MS, and JA collaborated in the interpretation of results, discussion, and review of this manuscript. AL, JK, WD, JA, and LR drafted the manuscript. All the authors approved the final manuscript and agreed to be responsible for the content of this study.

## Conflict of Interest

The authors declare that the research was conducted in the absence of any commercial or financial relationships that could be construed as a potential conflict of interest.

## Footnotes

1 https://bitbucket.org/izhbannikov/seqyclean

2 http://www.ncbi.nlm.nih.gov/tools/vecscreen/univec/

3 www.pantherdb.org
