# Supplementary material for: Potential Biomarkers for Feed Efficiency-Related Traits in Nelore Cattle Identified by Co-expression Network and Integrative Genomics Analyses
Source: Front Genet. 2020 Mar 4;11:189. doi: 10.3389/fgene.2020.00189 (PMC7064723; doi:10.3389/fgene.2020.00189)
Supplement: Supplementary file 1 [file Data_Sheet_1.zip › data_frontiers/Supplementary_Material02_Frontiers.pdf]

Potential biomarkers for feed efficiency-related traits in Nelore cattle identified by co-expression network and integrative genomics analyses

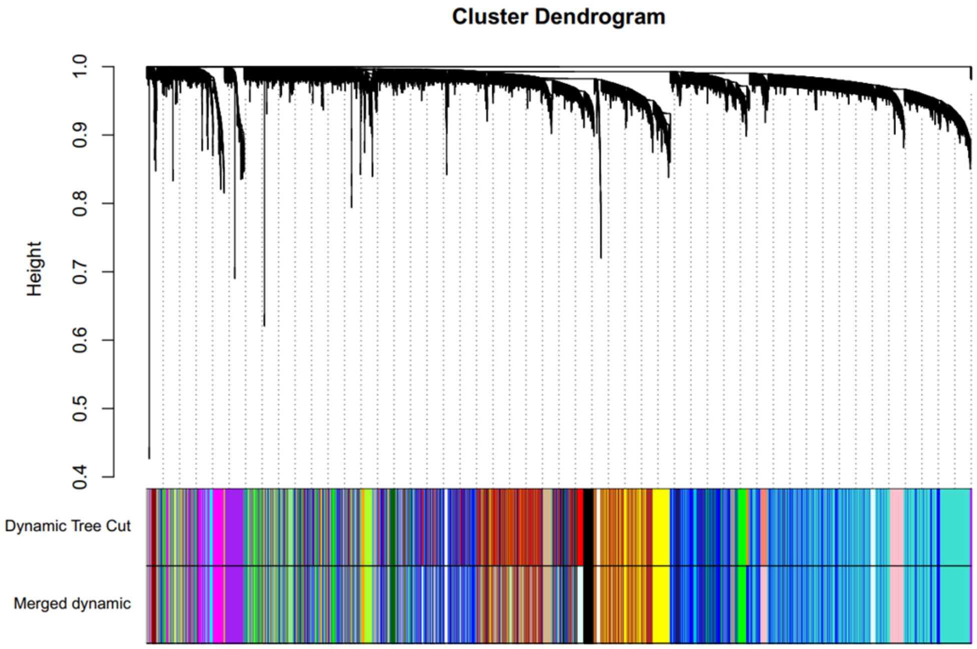

Supplementary Figure S1. Clustering dendrogram of genes in modules obtained by Weighted Gene Co-expression Network Analysis of *Longissimus thoracis* RNA-Seq samples from 180 Nelore (*Bos indicus*).

MTA- Module Trait Association

	BW	MBW	ADG	DMI	FCR	FE	KI	RGR	RFI
MEdarkred	-1e-05 (1)	-9e-05 (0.9)	-0.02 (0.3)	1e-04 (1)	0.006 (0.3)	-0.2 (0.3)	-2 (0.3)	-0.3 (0.1)	0.005 (0.6)
MElightgreen	2e-05 (0.9)	1e-04 (0.9)	-3e-04 (1)	0.004 (0.5)	0.002 (0.7)	-0.1 (0.6)	-0.3 (0.9)	-0.08 (0.7)	0.008 (0.3)
MEcyan	-1e-04 (0.5)	-7e-04 (0.5)	-4e-05 (1)	-4e-04 (0.9)	-6e-05 (1)	-0.03 (0.9)	0.2 (0.9)	0.1 (0.5)	0.004 (0.6)
MEdarkgreen	-1e-04 (0.4)	-8e-04 (0.4)	0.007 (0.8)	0.002 (0.7)	-0.002 (0.7)	-0.06 (0.8)	0.9 (0.7)	0.2 (0.4)	0.01 (0.2)
MEpink	-7e-05 (0.7)	-5e-04 (0.6)	-0.002 (0.9)	0.004 (0.4)	0.003 (0.6)	-0.2 (0.4)	0.1 (0.9)	0.1 (0.6)	0.01 (0.1)
MEturquoise	-1e-04 (0.5)	-6e-04 (0.5)	0.01 (0.7)	0.002 (0.7)	-0.001 (0.8)	9e-04 (1)	2 (0.5)	0.3 (0.1)	0.009 (0.3)
MEdarkturquoise	-5e-05 (0.8)	-3e-04 (0.8)	0.03 (0.2)	0.004 (0.5)	-0.008 (0.1)	0.2 (0.5)	3 (0.2)	0.4 (0.1)	0.007 (0.4)
MEmidnightblue	-8e-05 (0.6)	-5e-04 (0.6)	0.01 (0.5)	0.004 (0.4)	-0.004 (0.4)	0.002 (1)	2 (0.4)	0.2 (0.4)	0.01 (0.2)
MElightyellow	1e-04 (0.4)	8e-04 (0.4)	0.03 (0.2)	0.003 (0.6)	-0.007 (0.2)	0.2 (0.3)	2 (0.3)	0.4 (0.08)	-0.004 (0.6)
MEgreen	3e-04 (0.09)	0.002 (0.08)	0.03 (0.3)	0.01 (0.06)	-0.002 (0.8)	-0.1 (0.6)	0.7 (0.8)	-0.01 (1)	0.008 (0.4)
MEblack	-1e-04 (0.5)	-7e-04 (0.5)	0.03 (0.2)	-7e-04 (0.9)	-0.008 (0.2)	0.3 (0.2)	3 (0.1)	0.5 (0.02)	-7e-04 (0.9)
MElightcyan	-8e-05 (0.6)	-4e-04 (0.6)	0.04 (0.09)	0.001 (0.8)	-0.01 (0.04)	0.3 (0.1)	4 (0.05)	0.5 (0.01)	-4e-04 (1)
MEroyalblue	1e-04 (0.5)	6e-04 (0.5)	0.03 (0.2)	0.005 (0.4)	-0.006 (0.3)	0.2 (0.5)	3 (0.2)	0.3 (0.2)	0.001 (0.9)
MEblue	7e-05 (0.7)	4e-04 (0.7)	0.02 (0.3)	0.006 (0.3)	-0.003 (0.5)	-0.02 (0.9)	2 (0.5)	0.3 (0.3)	0.008 (0.4)
MEgrey60	9e-05 (0.6)	5e-04 (0.6)	0.04 (0.1)	0.006 (0.3)	-0.005 (0.4)	0.1 (0.6)	3 (0.2)	0.4 (0.07)	0.004 (0.6)
MEtan	-2e-04 (0.3)	-9e-04 (0.4)	0.03 (0.2)	-0.003 (0.5)	-0.01 (0.04)	0.4 (0.05)	4 (0.07)	0.5 (0.02)	-0.006 (0.5)
MEbrown	-4e-05 (0.8)	-2e-04 (0.8)	0.05 (0.05)	-0.001 (0.8)	-0.01 (0.01)	0.5 (0.02)	5 (0.03)	0.6 (0.01)	-0.009 (0.3)
MEyellow	-5e-05 (0.8)	-3e-04 (0.8)	0.06 (0.02)	-0.002 (0.7)	-0.02 (0.002)	0.6 (0.005)	6 (0.01)	0.6 (0.005)	-0.01 (0.2)
MEwhite	2e-04 (0.3)	0.001 (0.3)	0.02 (0.3)	0.004 (0.4)	-2e-04 (1)	0.07 (0.7)	1 (0.5)	0.3 (0.2)	-0.003 (0.8)
MEdarkgrey	6e-05 (0.7)	4e-04 (0.7)	0.007 (0.8)	-4e-04 (0.9)	-4e-04 (0.9)	0.05 (0.8)	0.2 (0.9)	0.2 (0.4)	-0.005 (0.6)
MEgreenyellow	4e-05 (0.8)	2e-04 (0.8)	-0.02 (0.5)	0.002 (0.8)	0.007 (0.2)	-0.2 (0.4)	-1 (0.5)	-0.09 (0.7)	0.005 (0.6)
MEorange	-7e-05 (0.7)	-4e-04 (0.6)	0.02 (0.4)	0.003 (0.6)	2e-04 (1)	0.1 (0.5)	3 (0.2)	0.4 (0.09)	0.007 (0.4)
MEmagenta	3e-05 (0.8)	2e-04 (0.8)	-0.01 (0.7)	-0.002 (0.7)	2e-04 (1)	-0.07 (0.8)	-2 (0.4)	-0.2 (0.3)	-0.006 (0.5)
MEpurple	-5e-05 (0.8)	-3e-04 (0.8)	3e-04 (1)	-0.007 (0.2)	-0.01 (0.07)	0.3 (0.2)	0.3 (0.9)	-0.06 (0.8)	-0.02 (0.05)
MEgrey	1e-04 (0.4)	9e-04 (0.3)	-0.02 (0.4)	0.003 (0.6)	0.007 (0.2)	-0.3 (0.1)	-3 (0.1)	-0.6 (0.006)	0.004 (0.7)

Supplementary Figure S2. Gene expression modules associated with feed efficiency related-traits (Body weight (BW), metabolic body weight (MBW), average daily gain (ADG), dry matter intake (DMI), food conversation ratio (FCR), feed efficiency ratio (FE), Kleiber index (KI), relative growth ratio (RGR), residual feed intake(RFI)). The coefficients from the linear model are given on the top in each cell of the matrix and the p-values are shown in parenthesis. Modules significantly associated with feed efficiency-related traits ($p < 0.05$) are shown in red boxes above.

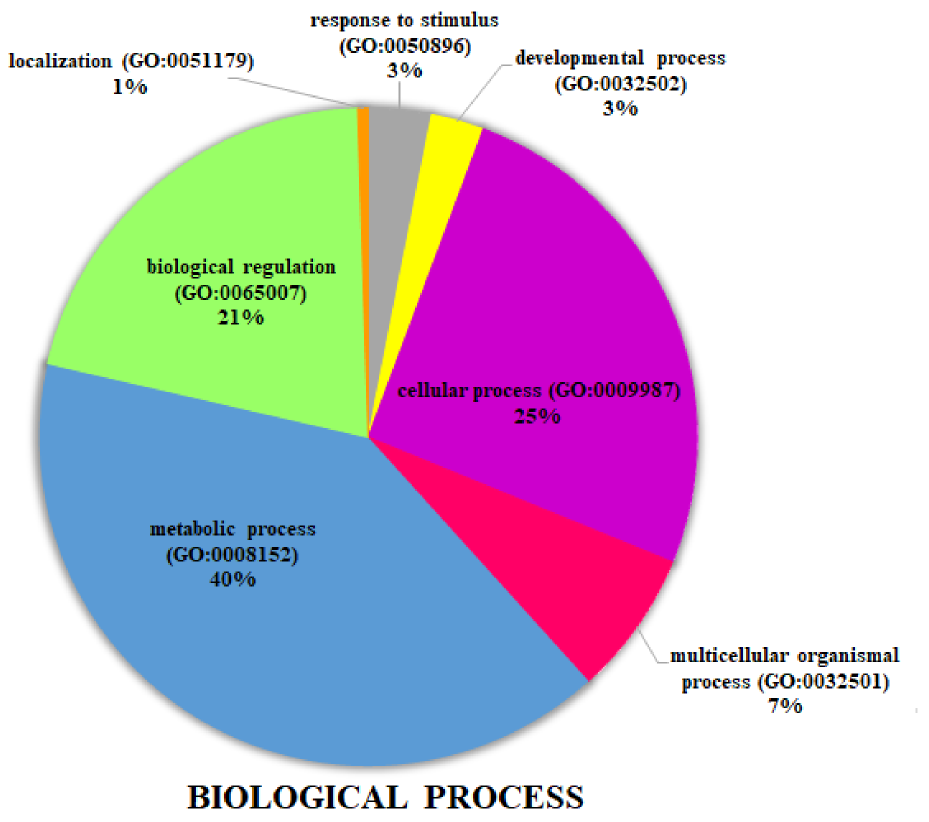

Supplementary Figure S3. The categorization of ninety-one transcription factors (TFs) (TFs interacting with hub genes belonging to at least two modules associated with feed efficiency related-traits) performed by PANTHER. The TFs were classified according to biological processes.
